# Cleaning Teeth

**Published:** 1888-02

**Authors:** 


ARTICLE IV.

### CLEANING TEETH.

Salivary calculus and stains on the teeth, at times, when the mouth is opened, will disgust the beholder, and frequently prevents the formation of a favorable opinion of the person, who is so neglectful of his appearance. Such disfigurements are more noticeable in the mouths of women and girls than in men, on account of the absence of a beard, which often conceals the teeth of men. Nothing adds so much to personal appearance as a clean set of teeth. How necessary it is, then, that dentists should, as examples to their patients, have clean teeth. Many, however, are unmindful of the fact that cleanliness begins at the fountain head. Teaching and preaching has little effect on a careless patient unless the dentist can exhibit spotless ivories. All of the preceding is only a prelude to a few remarks on the operative procedures relative to cleansing a set of teeth for a patient. The teeth of many people, from neglect, are found more or less disfigured by salivary deposits and stains. The gums are detached at the necks, ragged on the edges and bleed easily. Pus may ooze on pressure from between the teeth and gums. Now, if a patient has from twenty-six to thirty-two teeth, all more or less covered by deposits and agglutinized mucus, food, etc., it is improbable that a dentist, be he ever so expert, could properly remove *all* extraneous matter and thoroughly cleanse a set of teeth in one sitting, even of two or three hours duration. Our own practice is to syringe the mouth with tepid water, generally adding thirty or forty drops of a ten per cent. solution of resorcin to four ounces of water. The deposits are then removed from every tooth, beginning with the third molar, coming forward to the central incisor. This is done both above and below. The deposits are all removed with a pushing motion, save in a few instances, whereit is possible by the pulling movement to detach the concretion without tearing or wounding the gingival margin of the gum. It will be found, by a little practice, that this is the most effective method of operating, supposing that properly shaped instruments are used. It also saves time, produces less laceration of the gums and by consequence less interference in operating on account of the slight loss of blood. All stains are then removed from the teeth by the use of wooden, felt, moose-hide, leather or rubber points. These should be charged with levigated pumice, powdered Arkansas stone, oxide of tin and finely prepared chalk. During the whole cleaning process the only water used in the mouth of the patient should be injected from the syringe, as it is more efficient and saves time, and the jet of water more certainly removes the fine fragments of calculus and grains of powder than ordinary rinsing with water from a goblet. Fine brushes, which are revolved by the engine, are useful in the polishing process after the calculus has been removed. *Avoid the use of mineral acids in any form for cleaning teeth.—The Dental Review.*

